# Frequency of Pathogenic Germline Mutations in Early and Late Onset Familial Breast Cancer Patients Using Multi-Gene Panel Sequencing: An Egyptian Study

**DOI:** 10.3390/genes14010106

**Published:** 2022-12-29

**Authors:** Auhood Nassar, Abdel-Rahman N. Zekri, Mahmoud M. Kamel, Mostafa H. Elberry, Mai M. Lotfy, Mohamed G. Seadawy, Zeinab K. Hassan, Hany K. Soliman, Ahmed M. Lymona, Amira Salah El-Din Youssef

**Affiliations:** 1 Virology and Immunology Unit, Cancer Biology Department, National Cancer Institute, Cairo University, Cairo 11976, Egypt; 2 Clinical Pathology Department, National Cancer Institute, Cairo University, Cairo 11976, Egypt; 3 Baheya Centre for Early Detection and Treatment of Breast Cancer, Giza 3546211, Egypt; 4 Biological Prevention Department, Chemical Warfare, 4.5 km Suez-Cairo Rd, Almaza, Cairo 11351, Egypt; 5 Surgical Oncology Department, National Cancer Institute, Cairo University, Cairo 11976, Egypt

**Keywords:** multi-gene panel sequencing, familial breast cancer, germline mutations, *BRCA1*, *BRCA2*, precision oncology

## Abstract

Background: Precision oncology has been increasingly used in clinical practice and rapidly evolving in the oncology field. Thus, this study was performed to assess the frequency of germline mutations in early and late onset familial breast cancer (BC) Egyptian patients using multi-gene panel sequencing to better understand the contribution of the inherited germline mutations in BC predisposition. Moreover, to determine the actionable deleterious mutations associated with familial BC that might be used as biomarker for early cancer detection. Methods: Whole blood samples were collected from 101 Egyptian patients selected for BC family history, in addition to 50 age-matched healthy controls. A QIAseq targeted DNA panel (human BC panel) was used to assess the frequency of germline mutations. Results: A total of 58 patients (57.4%) out of 101 were found to have 27 deleterious germline mutations in 11 cancer susceptibility genes. Of them, 32 (31.6%) patients carried more than one pathogenic mutation and each one carried at least one pathogenic mutation. The major genes harboring the pathogenic mutations were: *ATM*, *BRCA2*, *BRCA1*, *VHL*, *MSH6*, *APC*, *CHEK2*, *MSH2*, *MEN1*, *PALB2*, and *MUTYH*. Thirty-one patients (30.6%) had *BRCA2* mutations and twenty (19.8%) had *BRCA1* mutations. Our results showed that exon 10 and exon 11 harbored 3 and 5 mutations, respectively, in *BRCA1* and *BRCA2* genes. Our analysis also revealed that the *VHL* gene significantly co-occurred with each of the *BRCA2* gene (*p* = 0.003, event ratio 11/21), the *MSH2* gene (*p* = 0.01, 4/10), the *CHEK2* gene (*p* = 0.02, 4/11), and the *MSH6* gene (*p* = 0.04, 4/12). In addition, the *APC* gene significantly co-occurred with the *MSH2* gene (*p* = 0.01, 3/7). Furthermore, there was a significant mutually exclusive event between the *APC* gene and the *ATM* gene (*p* = 0.04, 1/36). Interestingly, we identified population specific germline mutations in genes showing potentials for targeted therapy to meet the need for incorporating precision oncology into clinical practice. For example, the mutations identified in the *ATM*, *APC*, and *MSH2* genes. Conclusions: Multi-gene panel sequencing was used to detect the deleterious mutations associated with familial BC, which in turns mitigate the essential need for implementing next generation sequencing technologies in precision oncology to identify cancer predisposing genes. Moreover, identifying DNA repair gene mutations, with focus on non-BRCA genes, might serve as candidates for targeted therapy and will be increasingly used in precision oncology.

## 1. Introduction

According to the World Health Organization (WHO), 2.3 million women worldwide were diagnosed with BC in 2020 [1]. In Egypt, BC is the most prevalent malignancy in females, accounting for 32% of all cancers in the Egyptian population, with an estimated three-fold increase forecasted in 2050 [2]. Current advances in BC treatment significantly improved BC patients’ survival, especially those diagnosed early [3,4]. However, the effectiveness of prevention and treatment will remain restricted without a complete understanding of the underlying mechanism and pathogenesis [3]. Due to the increased use of genomic profiling to identify genetic variants with possible diagnostic and prognostic implications, precision oncology is rapidly evolving in the oncology field [5]. Germline genetic information can also affect the targeted cancer therapy choices [6]. For example, patients with germline pathogenic variants in DNA repair genes are potential candidates for PARP inhibitors [7]. In addition, identification of pathogenic germline variants can identify at-risk relatives and help in cancer prevention and early detection [8]. Thus, it is crucial to implement next generation sequencing technologies in precision oncology.

Germline mutations in one or both *BRCA* genes were associated with a high risk of BC development and incidence of other malignancies [9,10]. *BRCA1* and *BRCA2* genes have been reported to be the most frequently mutated genes associated with familial BC [11]. Approximately 7% of BC is estimated to be primarily due to germline mutations in the BRCA1/2 genes. The cumulative BC risk is reported to be 72% and 69%, respectively, for BRCA1 and BRCA2 mutation carriers [12]. Women who carry deleterious *BRCA1*/*2* mutations are recommended to undergo prophylactic risk-reducing surgery to decrease cancer-related mortality. In addition, they are also recommended to do BC screening by magnetic resonance imaging (MRI) [13]. In addition to *BRCA1/2* genes, there are other genes in which mutations are associated with familial BC, such as *PALB2*, *CHEK2*, *ATM*, *MSH2*, *MSH6*, and *MUTYH* genes [14]. Mutations of these genes have been previously reported to be involved in the homologous recombination (HR) pathway, DNA damage response pathway, and mismatch recognition pathway [15,16]. Thus, dysfunction of these pathways could induce genomic instability, which in turn drives cancer development [17]. Moreover, the National Comprehensive Cancer Network (NCCN) guidelines for hereditary BC genetic risk assessment recommends genetic evaluation of the *ATM*, *CHEK2*, *NBN*, *NF1*, and *PALB2* genes beside the *BRCA1/2* genes [18]. Although, searches for BC predisposition genes have been made in the past decade, more studies are still needed to identify additional BC susceptibility genes to find their association with BC risk assessment [19,20].

Since the advent of next-generation sequencing (NGS), multi-gene testing panels have been increasingly applied to detect genetic mutations that may be associated with increased BC risk [20]. However, very limited studies have addressed the frequency of germline mutations in BC susceptibility genes among Egyptian patients with BC family history, such as Saied et al. [21] and Kim et al. [22]. Thus, in the current study and to the best of our knowledge, we were the first to assess the frequency of deleterious germline mutations in familial BC Egyptian patients using a multi-gene panel sequencing of 93 genes, to better understand the contribution of the inherited germline mutations in the predisposition of BC in an Egyptian BC cohort selected for BC family history.

## 2. Methods

### 2.1. Patient Selection

This study was conducted on 101 familial BC patients and 50 age-matched healthy controls. Samples were obtained from patients who underwent surgical resection for primary BC at the Egyptian National Cancer Institute (NCI). Patients were selected if they had: (a) BC at any age of diagnosis, and (b) with BC family history (at least one first- or second-degree relative). Patients were classified according to their age, histological type, histological grade, and hormone receptor status (estrogen receptor (ER), progesterone receptor (PR), and Her2-neu). All the clinicopathological data of the studied patients were collected from the clinical records at the NCI. All protocols and procedures were approved by the Institutional Review Board (IRB number: IRB00004025; approval number: 201617043.3) of NCI, Cairo, Egypt. Written informed consent was obtained from all patients during their enrolment in this study.

### 2.2. DNA Extraction

Whole blood (10 cm) was collected from each of the 101 familial BC female patients and 50 age-matched healthy controls. DNA was then isolated using QIAamp DNA Blood Mini Kit (Qiagen, Hilden, Germany: Cat. No.51104). Then, DNA was quantified using Qubit^®^ 3.0 Fluorometer (Cat. No, Q33216, Thermofisher Scientific Inc., Waltham, MA, USA) and Qubit™ dsDNA HS assay kit (Cat. No. Q32854, Thermofisher Scientific Inc., Waltham, MA, USA).

### 2.3. NGS Assay

We used QIAseq targeted DNA panel (human breast cancer panel) (Cat. No. 333505, Qiagen, Germany). This panel included 93 BC specific genes (*ACVR1B*, *AKT1*, *APC*, *AR*, *ATM*, *ATR*, *AXIN2*, *BAP1*, *BARD1*, *BLM*, *BMPR1A*, *BRCA1*, *BRCA2*, *BRIP*, *CASP8*, *CBFB*, *CCND1*, *CDH1*, *CDK4*, *CDK6*, *CDKN2A*, *CHEK2*, *CSMD1*, *CTNNB1*, *DIRAS3*, *EGFR*, *EP300*, *EPCAM*, *ERBB2*, *ERBB3*, *ERCC4*, *ESR1*, *EXOC2*, *EXT2*, *FAM175A*, *FANCC*, *FBXO32*, *FGFR1*, *FGFR2*, *GATA3*, *GEN1*, *HERC1*, *HOXB13*, *IRAK4*, *ITCH*, *KMT2C*, *KRAS*, *MAP2K4*, *MAP3K1*, *MDM2*, *MED12*, *MEN1*, *MLH1*, *MRE11A*, *MSH2*, *MSH6*, *MUC16*, *MUTYH*, *MYC*, *NBN*, *NCOR1*, *NEK2*, *NF1*, *PALB2*, *PALLD*, *PBRM1*, *PCGF2*, *PIK3CA*, *PIK3R1*, *PMS1*, *PMS2*, *PPM1L*, *PTEN*, *PTGFR*, *RAD50*, *RAD51*, *RAD51C*, *RAD51D*, *RB1*, *RET*, *SEPT9*, *SMAD4*, *SMARCA4*, *STK11*, *SYNE1*, *TGFB1*, *TP53*, *TRAF5*, *VHL*, *WEE1*, *XRCC2*, *XRCC3*, and *ZBED4*).

The NGS libraries were constructed according to the manufacturer’s instructions. Then, the fragment size and concentration were checked using QIAxcel DNA high-resolution kit (Cat No. 929002, Qiagen, Hilden, NRW, Germany). Then, libraries were quantified using QIAseq Library Quant Assay Kit (Cat No. 333304, Qiagen, Hilden, NRW, Germany). The Ion PI Hi-Q Chef Kit (Cat. No. A27198, Thermo Fischer Scientific Inc., Waltham, MA, USA) was used for template preparation and the Ion Proton Sequencing 200 Kit v2 (Cat. No. 4485149, Thermo Fischer Scientific Inc., Waltham, MA, USA) was used for sequencing on the Ion Proton Platform.

### 2.4. Bioinformatics Analysis

The Ion Torrent Suite was used for base calling, alignment, and variant analysis. The run was only successful if the depth was larger than 100× and the coverage was more than 95% of the target regions. The read analysis workflow for reads from QIAseq UMI-based targeted DNA enrichment began with read processing steps that removed the exogenous sequences such as PCR and sequencing adapters and UMI (unique molecular index), then identified the UMI sequence and appended it to the read identifier for downstream analyses and, after that, removed short reads that lack enough endogenous sequence for mapping to the reference genome (hg19/GRCH37). Then, the reads were aligned to the human reference genome (version hg19). The bases with base quality less than Q20 were trimmed and the reads of low-quality were excluded. We used the QIAGEN Gene Globe Data Analysis Center and the Torrent Suite Variant Caller to call the germline variants. For variant annotation, we used the QIAGEN GeneGlobe Data Analysis Center including population databases.

Synonymous, non-exonic, and splicing variants were filtered out. To further assess that the remaining variants were cancer-specific, we used the variants of the control samples to filter out normal inherited polymorphism. Functional consequences of the identified variants were predicted using Sift [23], PolyPhen-2 [24], and CADD [25] tools.

Variant information was obtained using the dbSNP database (http://www.ncbi.nlm.nih.gov/projects/SNP, accessed on 1 October 2022), the Human Gene Mutation Database (HGMD), the 1000 Genome project, and the ClinVar database (http://www.ncbi.nlm.nih.gov/clinvar/), accessed on 18 June 2022. Mutations were classified according to American College of Medical Genetics and Genomics (ACMG) recommendations [26] into benign, likely benign, variants of uncertain significance, likely pathogenic (LP), and pathogenic (P). In this study, we considered the variant to be pathogenic (deleterious) if it was classified as being P or LP.

### 2.5. Statistical Analysis

The analysis of clinicopathological characteristics between deleterious mutation carriers and non-carriers was performed using the Pearson’s chi-square test. Two-sided *p*-value <0.05 was considered statistically significant. Mutually exclusive and co-occurring events between gene pairs harboring P and LP mutations were identified using pair-wise Fisher’s exact test and the CoMet ExactTest algorithm. The event was considered statistically significant if *p* value <0.05. We performed data visualization using R package (version 4.2).

The lollipop plots and oncoplot were visualized using Mutation Annotation Format tools (maftools), R/Bioconductor package (https://www.r-project.org/), accessed on 26 June 2022.

## 3. Results

### 3.1. Patient Features

All the studied patients were ER and/or PR positive and Her-2 negative (only 2 patients were Her-2 positive). No triple negative BC patients were enrolled in this study. The characteristics of patients with and without pathogenic mutations are summarized in Table 1. In our study, the age of BC onset ranged from 21 years to 53 years. The mean age at diagnosis was 37.3 years. Of all 101 patients, 58 patients (57.4%) were found to have P/LP germline mutations of cancer susceptibility genes and 43 patients (42.5%) were not found to carry P/LP mutations. The BC grade, tumor type, and age at diagnosis were not different between the two groups, (*p* = 0.52, *p* = 0.41, *p* = 0.88) respectively. However, there was a significant difference between *BRCA1/2* positive patients carrying P/LP mutations and those without (*p* < 0.001). The proportion of deleterious mutations varied among BC patients diagnosed at age of ≤40 years and >40 years, and among the *BRCA1/2* positive and *BRCA1/2* negative patients. The deleterious mutations were found in 41 of 58 patients (70.6%) who had BC at age ≤40 years, and 17 of 58 patients (29.3%) who were diagnosed with BC at age >40 years old. The highest proportion of P/LP mutations was found in BC patients who were diagnosed at ≤40 years old and the lowest proportion was found in BC patient who carried *BRCA1/2* negative gene mutation.

### 3.2. NGS Dataset Description

**Figure 1:**
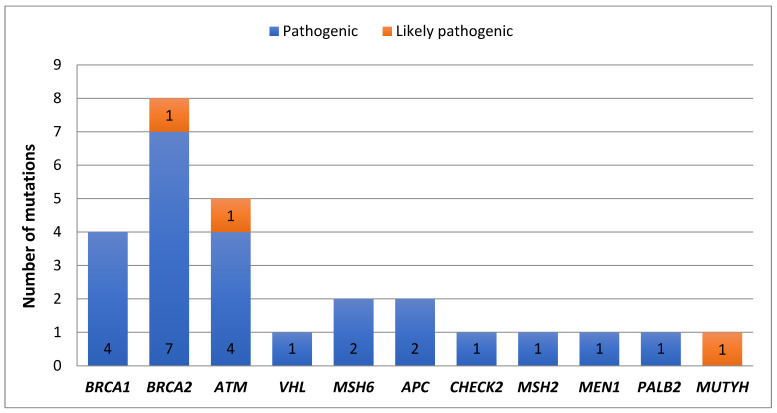
Distribution of pathogenic and likely pathogenic mutations identified with multi-gene panel.

Our NGS analysis revealed 426 candidate variants in 93 genes. The maximum number of variants per individual sample was 6, and the median number of variants per sample was 24. The identified candidate variants were classified into 163 benign, 63 likely benign, 31 variants of uncertain significance (VUS), 25 pathogenic (P), two likely pathogenic (LP), and 142 variants that were not reported in the ClinVar database. The distribution of pathogenic and likely pathogenic mutations identified with the multi-gene panel is shown in Figure 1. The mean depth of coverage of the identified variants ranged from 500 to 1000× in the studied patients. The whole set of the identified variants is in sheet 1 in the Supplementary File S1 and also the diagrammatic representation of the identified InDels is in sheet 2 in the Supplementary File S1.

### 3.3. Frequency of Pathogenic Mutations Identified in This Cohort

**Figure 2:**
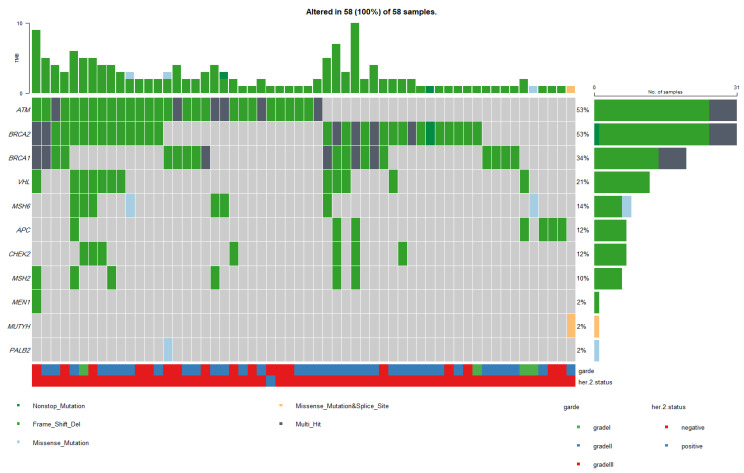
Oncoplot displays the pathogenic germline mutations identified in 58 patients out of 101.

In the current study, the pathogenic mutations identified with multi-gene panel sequencing are listed in Table 2. Sequencing analysis showed 25 pathogenic mutations and 2 likely pathogenic mutations in 58 patients out of 101 as shown in Figure 2. Our analysis revealed that each patient in the altered 58 carried at least one pathogenic mutation. Furthermore, 32 (31.6%) patients carried more than one pathogenic mutation. The major genes harboring the pathogenic mutations were: *ATM*, *BRCA2*, *BRCA1*, *VHL*, *MSH6*, *APC*, *CHEK2*, *MSH2*, *MEN1*, *PALB2*, and *MUTYH*. Notably, 31 (30.6%) patients carried pathogenic mutations in the *ATM* gene; 14 (13.8%) patients out of them co-occurred with the *BRCA2* gene. Moreover, the most frequent pathogenic mutation in this study was *ATM* c.8432delA, which was identified in 24 (23.7%) patients.

### 3.4. Frequency of BRCA1/2 and Other DNA Repair Genes Identified in This Cohort

Our results showed that among 101 patients, 40 (39.6%) carried *BRCA1* and *BRCA2* pathogenic mutations; 20 (19.8%) were *BRCA1* and 31 (30.6%) were *BRCA2*. Most of the altered genes in this study were key DNA repair genes including *ATM*, *BRCA1*, *BRCA2*, *MSH6*, *CHEK2*, *MSH2*, *PALB2*, and *MUTYH*. Thus, to know the prevalence of the deleterious germline mutations in the identified DNA repair genes and to analyze the mutation rates among the different tumor grades (grade I, II, and III), we listed the identified DNA repair genes according to their roles in the process of DNA damage repair and BC carcinogenesis [11]. No significant differences in the mutation rates were observed, as shown in Table 3.

### 3.5. Frequency of Non-BRCA Genes in BRCA1/2 Negative Patients

Our analysis showed that 18 patients (17.8%) were *BRCA*-negative and carried pathogenic mutations in non-*BRCA* genes. The identified mutant non-*BRCA* genes were: *ATM* in 12 patients (11.8%), *MSH6* in 3 patients (2.9%), *APC* in 4 patients (3.9%), and *VHL*, *CHEK2*, *MSH2*, and *MUTYH* genes, each, in 1 patient (0.9%). We found one participant who carried two distinct mutations, which were *VHL* c.444delT and *APC* c.3814delT. Moreover, another identified participant carried another two distinct mutations, which were *MSH6* c.3312delT and *MSH2* c.2647delA.

### 3.6. Patients with Pathogenic Germline Mutations in Single and Multiple Genes

Table 4 lists the patient numbers with pathogenic germline mutations in single and multiple genes. We identified 26 patients who carried pathogenic mutations only in one gene. In addition, other patients carried pathogenic mutations in more than one gene (Table 4). The maximum number of altered genes per individual sample was six genes. Our analysis also revealed that patients with multi-hit mutations within the same gene were identified only in the *BRCA1*, *BRCA2*, and *ATM* genes. The List of variants of uncertain significance and variants of conflicting interpretation of pathogenicity identified in this study is shown in Table 5.

### 3.7. The Distribution of Pathogenic Gene Mutations in Exon Regions and Protein Domains

**Figure 3:**
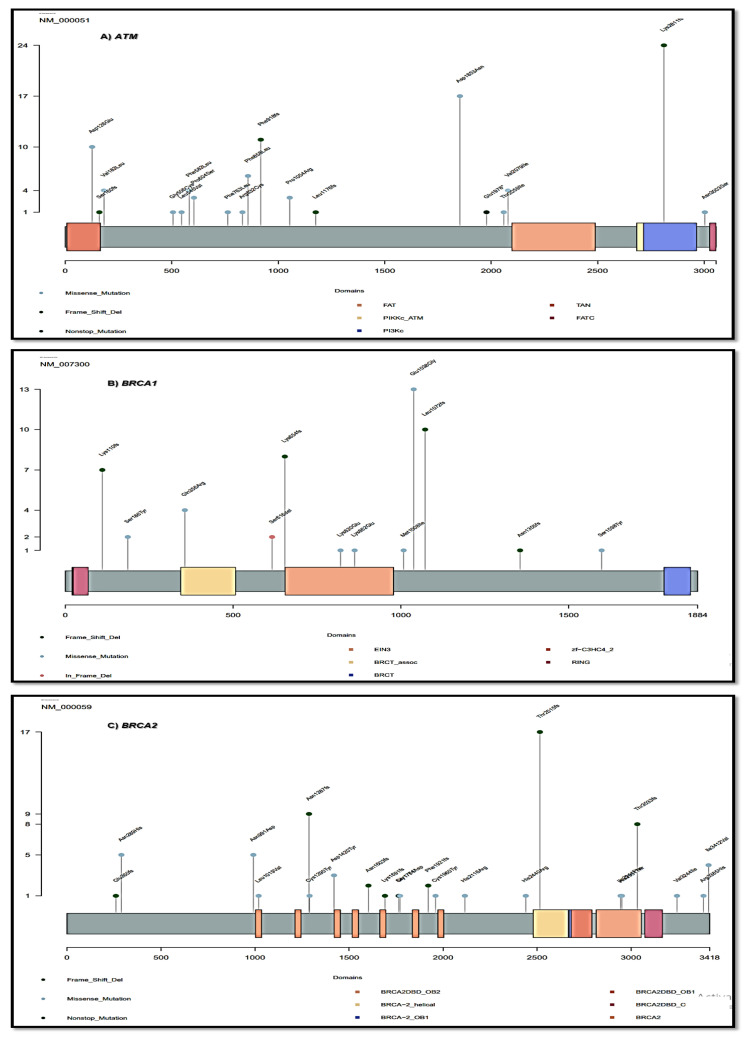
Schematic representation showing the position of the detected germline mutations in (**A**) *ATM*, (**B**) *BRCA1*, and (**C**) *BRCA2* genes. The mutations are colored with respect to their type.

Our results showed that exon 10 of the *BRCA1* gene and exon 11 of the *BRCA2* gene possessed a high number of mutations: three mutations in the *BRCA1* gene and five mutations in the *BRCA2* gene. The most affected two protein domains in the *BRCA2* gene are BRCA2-helical domain and BRCA2-DBD-OB2 (BRCA2 DNA binding domain). Herein, in exon 15, we identified one pathogenic BRCA2-helical mutation (p.Thr2515fs) in 17 patients. In addition, another pathogenic mutation (p.Thr3033fs), in exon 23, was located in the BRCA2-DBD-OB2 domain and it was identified in 8 patients. In the *ATM* gene, exon 58 was the most affected; it harbored one pathogenic mutation (p.Lys2811fs) in 24 patients (23.7%). This mutation resides within the PI3Kc (phosphatidylinositol 3-kinase catalytic domain) of the *ATM* gene. Figure 3A–C shows the schematic representation of the detected germline mutations in the *ATM*, *BRCA1*, and *BRCA2* genes.

### 3.8. Mutually Exclusive and Co-Occurring Events between Gene Pairs with Deleterious Mutations

**Figure 4:**
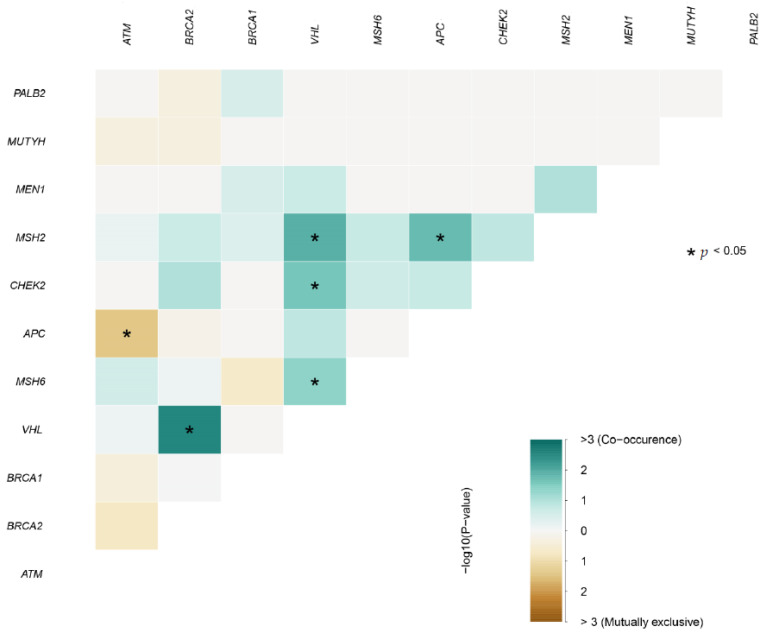
Co-occurrence plot shows the gene pairs with co-occurring and mutually exclusive events. Degree of significance indicated in the legend, with only results of the genes harboring deleterious mutation shown. Genes: *PALB2* [1]; *MUTYH* [1]; *MEN1* [1]; *MSH2* [6]; *CHEK2* [7]; *APC* [7]; *MSH6* [8]; *VHL* [12]; *BRCA2* [31]; *ATM* [31].

Figure 4 shows gene pairs with co-occurring and mutually exclusive events. There were five significant co-occurrence events and one significant mutually exclusive event between gene pairs harboring P and LP mutations identified in our study; the *VHL* gene co-occurred with the *BRCA2* gene (*p* = 0.003, event ratio 11/21), the *MSH2* gene (*p* = 0.01, 4/10), the *CHEK2* gene (*p* = 0.02, 4/11), and the *MSH6* gene (*p* = 0.04, 4/12). In addition, the *APC* gene co-occurred with the *MSH2* gene (*p* = 0.01, 3/7). Furthermore, there was a mutually exclusive event between the *APC* gene and the *ATM* gene (*p* = 0.04, 1/36).

## 4. Discussion

Identifying the causal mutations of hereditary tumors not only directs the tumor surveillance and preventive strategies but also impacts the targeted treatment and prognosis in mutation carriers [27]. Thus, to meet the need for precise diagnosis and treatment, identifying the population-specific variant is crucial in incorporating accurate genetic testing of *BRCA* genes into clinical practice in definite populations and ethnic groups [28]. The use of NGS in cancer genetics and the diagnostics of hereditary cancers have been revolutionized in the last decade [29]. Hereby, we used NGS in our study to assess the germline mutation frequencies in early and late onset familial BC patients using multi-gene panel sequencing to better understand germline mutations’ role in BC predisposition. Among 101 Egyptian BC patients, a total of 27 deleterious mutations were detected. Of them, 12 mutations were detected in *BRCA1/2* genes and 15 were detected in non-BRCA genes.

Few relevant studies have been conducted to address the frequency of germline mutations in familial Egyptian BC patients, and they were only to investigate *BRCA1/2* germline profiling. For example, a recent study by Saied et al. investigated the frequency of *BRCA1/2* variants in patients with BC and their relatives [21]. Another study by Kim et al., in 2017, used whole exome sequencing to investigate genetic predisposition in five Egyptian families with BC. This study reported no pathogenic variants in *BRCA1/2* genes and other cancer predisposing genes which disagrees with our findings [22]. One possible explanation is that the use of targeted panel sequencing is superior to whole exome sequencing because it has higher read depth and coverage of target genes [30,31].

Mutations in DNA repair genes are commonly the underlying genetic cause of hereditary cancers [32]. *BRCA1* and *BRCA2* are key DNA repair genes that play a critical role in DNA double-strand breakage repair [11,33]. In the current study, we found that 40 patients had deleterious mutations in *BRCA1* and *BRCA2* genes, with a total prevalence of 39.6%, which is higher than that reported in a number of previous studies on familial BC patients in Middle East countries, such as Lebanon [34], Bahrain [35], and Saudi Arabia [36]. However, the prevalence of *BRCA1/2* deleterious mutations identified in this cohort (39.6%) is similar to that reported in a study conducted in Qatar on high risk BC patients [37]. On the other hand, when comparing our prevalence to that reported in North African countries, we also found a higher prevalence of *BRCA1/2* pathogenic mutations in the current studied Egyptian cohort. In a previous study on selected patients with BC and/or ovarian cancer from the south of Tunisia, the overall frequency of the BRCA1/2 germline mutations was 14.17%, which is lower than ours [38]. Another study in Morocco reported a 28% prevalence of *BRCA1/2* pathogenic mutations which is also lower than our percentage [39]. Additionally, a recent study in Algeria on hereditary breast and ovarian cancer families reported only 7 carriers of *BRCA1/2* pathogenic mutations out of 113 patients, representing 6.19%, which is lower than ours as well [40]. Our results reveal that the incident rate of *BRCA1/2* pathogenic mutations in familial BC patients in Egypt is higher than in North African populations.

Regarding *BRCA1* results, we identified four different deleterious mutations. We identified one *BRCA1* c.3214delC (p.Leu1072fs) pathogenic variant in 10 patients; this variant was reported by Wong et al. in Singapore to be inherited in Asian patients with features of hereditary breast and ovarian cancer [41]. This variant was also detected by Yang et al. among Malaysian BC patients in two different cases: the first one had a family history and was diagnosed at a very early age (23 years), and the other one had no family history and was diagnosed at 42 years old [42]. Furthermore, we also identified the c.1961delA (p.Lys654fs) *BRCA1* pathogenic variant in seven patients, and it was previously reported as a recurrent founder mutation in BC patients in different countries and worldwide [43,44,45,46]. On the other hand, we identified eight different deleterious mutations in the *BRCA2* gene. This c.3860delA (p.Asn1287fs) *BRCA2* pathogenic variant was previously reported in a Moroccan study in a patient suffering from ovarian cancer [39]; however, in this study, it was detected in nine patients with BC family history. Another identified *BRCA2* pathogenic variant (c.9097delA) in this study was previously identified in North African breast/ovarian cancer patients in Tunisia. One more c.4808delA (p.Asn1603fs) *BRCA2* pathogenic variant (rs397507743) was also reported as a recurrent founder mutation in the Brazilian population [43].

Interestingly, we identified one uncommon c.5291C > G BRCA2 pathogenic mutation (p.Ser1764*) that has been previously reported in five Slovene hereditary breast and ovarian cancer families [47]. Our findings revealed that familial BC Egyptian patients have high genetic variations, which reflect their different ethnic origins. Of note, mutations found in the *BRCA2* gene (29.6%) in this cohort were almost two times higher than those found in the *BRCA1* gene (14.8%), which is different from that reported in the literature [39,43,48,49]. Our findings suggest a higher *BRCA2* mutation burden in Egyptian patients with familial BC.

We identified key DNA repair genes in this study, including *ATM*, *BRCA1*, *BRCA2*, *MSH6*, *CHEK2*, *MSH2*, *PALB2*, and *MUTYH*. Our results are well-matched with Jalkh et al., who identified pathogenic mutations in *ATM*, *APC*, and *MSH2* genes in Lebanese familial BC patients [34]. In addition, they are also similar to those reported in a previous study on Korean hereditary BC patients that detected deleterious mutations in the *PALB2* and *CHEK2* genes [20]. Another Korean study by Shin et al. identified 35 patients (8%) with pathogenic mutations in the *CHEK2*, *MSH2*, and *MUTYH* genes in BC patients with clinical features of hereditary cancer syndrome [33]. Moreover, our findings come in agreement with those reported in two previous studies: the first one identified pathogenic mutations in *ATM*, *CHEK2*, and *PALB2* genes [50], and the second one identified pathogenic mutations in the *ATM*, *PALB2*, *CHEK2*, *MSH2*, *MSH6*, and *MUTYH* genes in Chinese patients with familial breast/ovarian cancer [48]. Germline alterations in DNA repair genes have been known as a contributing factor in the predisposition to hereditary cancer [51]. It was reported that most of the non-BRCA genes repeatedly detected in BC participate in DNA repair pathways. For example, DNA double-strand break repair variants in *PALB2*, *CHEK2*, *ATR*, *RAD51*, *RAD50*, and *ATM* genes were reported in BC [52]. In addition, variants in mismatch repair (MMR) genes, such as *MSH6*, *MSH2*, *PMS2*, and *MLH1*, and variants in genes participating in base excision repair, such as *MUTYH*, were also reported in BC [53,54]. The study of the mechanisms involved in DNA repair pathways has identified selective targets for therapy, such as PARP inhibitors which are currently approved for some BC cases. For example, Talazoparib that was approved by the FDA in October, 2018 for germline BRCA-mutated HER2-negative locally advanced or metastatic BC [55]. Moreover and besides PARP, there are other key components, such as *ATM* [56] and *MSH2* [57], with potential for targeted therapy. For instance, M3541, the *ATM* inhibitor currently used in clinical trials [58]. Thus, we consider that BC patients with germline mutations in DNA repair genes such as *PALB2*, *ATM*, and *CHEK2*, might benefit from PARP inhibitors. We also recommend more studies to focus on DNA repair gene mutations, including non-BRCA genes, to understand their role as selective markers for targeted therapy. In addition, studies are suggesting that tumors with DNA repair gene mutations are responsive to immunotherapy [59]. In BC, analysis of mutated DNA repair genes is critical to determine the reason for the high mutation load. Studies are revealing that there is a possible relationship between immunotherapy response and the altered DNA repair pathways that increase the tumor mutation burden (14). Thus, more research is needed to study if BC patients harboring DNA repair gene mutations could benefit from immunotherapy [59].

In addition, we identified 18 BRCA1/2-negative patients (17.8%) carrying deleterious mutations which were mainly in the *ATM*, *MSH6*, *APC*, *VHL*, *CHEK2*, *MSH2*, and *MUTYH* genes. This proportion is near to that reported by Maxwell et al. in their study on Caucasian and African American BC patients who were BRCA1/2 negative, which identified 11% of patients as carrying non-BRCA1/2 deleterious mutations [60]. The deleterious mutations were *ATM* (25.8%) compared to ours (11.8%), *CHEK2* (32.3%) compared to ours (0.9%), *MSH6* (3.2%) compared to ours (2.9%), and *MUTYH* (3.2%) compared to ours (0.9%). Additionally, our results were, to some extent, similar to a previous study on the Western population by Li et al., who detected 11.5% BRCA1/2-negative patients out of 660 familial BC patients carrying mutations in the *ATM*, *CHEK2*, and *PALB2* genes [61]. In addition, our proportion is higher than that reported in a recent study on Chinese patients with features of hereditary BC that found 6.8% of patients had non-BRCA1/2 mutations in the *ATM*, *CHEK2*, and *PALB2* genes [60]. However and disagreeing with our results, other studies reported a low frequency of deleterious mutations in BRCA1/2-negative patients with BC family history [20,62]. In BRCA1/2-negative patients, the most frequently detected deleterious non-BRCA1/2 mutations were *ATM* germline mutations, followed by *MSH6* and *APC*. Interestingly, *ATM*, a moderate-penetrance cancer susceptible gene, and *APC* genes were previously reported as key components showing potential for targeted therapy [63].

A co-mutation analysis was also performed to identify the significantly co-occurring or mutually exclusive gene pairs. Analysis was performed using the 11 genes harboring the pathogenic and likely pathogenic mutations. Five gene pairs were found to be significantly co-occurring. Of note, the *VHL* gene was found to co-occur with four other genes, suggesting its significant role in the predisposition of BC in Egyptian patients with family history.

To the best of our knowledge, this study is the first to describe multiple-gene panel sequencing in Egyptian patients with BC family history. We showed that the prevalence of *BRCA1* and *BRCA2* deleterious mutations (39.8%) is higher than in other populations. Additionally, we suggested a higher *BRCA2* mutation burden in familial BC Egyptian patients. Moreover, our data revealed that DNA repair gene mutations, with focus on non-BRCA genes, might serve as candidates for targeted therapy. The current study also reported the non-BRCA gene mutation status in familial BC patients for the first time in Egypt. In conclusion, our results contributed to the knowledge of germline variations in multiple cancer susceptible genes in familial BC Egyptian patients, mitigating the essential need for implementing high-throughput NGS technologies in precision oncology to identify cancer predisposing genes.

## Figures and Tables

**Table 1:** Characteristics of patients with and without pathogenic mutations.

Characteristic	Total (%) 101 (100)	P/LP Mutation Carriers	P/LP Mutation Non-Carriers	*p*-Value
**Number of patients**	**101 (100)**	**58**	**43**	
**BC Grade**				
I	**7 (6.9)**	**4**	**3**	
II	**67 (66.3)**	**36**	**31**	**0.52**
III	**27 (26.7)**	**18**	**9**	
**Tumor type**				
Invasive ductal carcinoma	**94 (93.06)**	**52**	**42**	
Invasive tubular carcinoma	**2 (1.98)**	**2**	**0**	**0.41**
Invasive lobular carcinoma	**4 (3.96)**	**3**	**1**	
micropapillary carcinoma	**1 (0.99)**	**1**	**0**	
**Age at diagnosis**				
BC diagnosis at ≤40 years	**72 (71.3)**	**41**	**31**	**0.88**
BC diagnosis at >40 years	**29 (28.7)**	**17**	**12**	
**BRCA1/2 status**				
**BRCA1/2 positive**	**40 (39.6)**	40 *	**0**	<0.001 **
**BRCA1/2 negative**	**61 (60.4)**	18 *	**43**	

BC: breast cancer; P: pathogenic; PL: likely pathogenic; MBC: metastatic breast cancer; *: mutations other than BRCA1/2; **; *p*-value is significant

**Table 2:** List of pathogenic and likely pathogenic mutations identified with multi-gene panel sequencing.

Gene	Position	dbSNP	Frequency	Type	Clinical Significance	Exon	HGVS.c	HGVS.p	Grade 1	Grade 2	Grade 3
*ATM*	Chr11:10821647	rs587782558	24	Indel	PV	57	c.8431_8432delAA	p.Lys2811fs	1	12	11
	Chr11:108139249	rs786202608	11	Indel	PV	18	c.2754delT	p.Phe918fs	-	8	3
	Chr11:108151842	rs730881302	1	Indel	PV	24	c.3526delC	p.Leu1176fs	-	1	-
	Chr11:108106541	rs587780624	1	Indel	LPV	5	c.478_482delTCTCA	p.Ser160fs	-	-	1
	Chr11:108183151	rs587779852	1	SNP (Stop gain)	PV	40	c.5932G > T	p.Glu1978 *	-	1	-
*BRCA2*	Chr13:32930667	rs80359657	17	Indel	PV	15	c.7543delA	p.Thr2515fs	2	12	3
	Chr13:32912345	rs80359406	9	Indel	PV	11	c.3860delA	p.Asn1287fs	-	7	2
	Chr13:32954022	rs397507419	8	Indel	PV	23	c.9097delA	p.Thr3033fs	-	4	4
	Chr13:32913558	rs80359479	1	Indel	PV	11	c.5073delA	p.Lys1691fs	-	1	-
	Chr13:32913295	rs80359466	2	Indel	PV	11	c.4808delA	p.Asn1603fs	-	1	1
	Chr13:32913783	rs397507778	1	SNP (Stop gain)	PV	11	c.5291C > G	p.Ser1764 *	-	1	-
	Chr13:32914250	rs80359534	2	Indel	PV	11	c.5763delT	p.Phe1921fs	-	2	-
	Chr13:32905146	rs75096777	1	Indel	PV	9	c.774_775delAA	p.Glu260fs	-	1	-
*BRCA1*	Chr17:41244333	rs80357923	10	Indel	PV	10	c.3214delC	p.Leu1072fs	-	9	1
	Chr17:41245586	rs80357522	8	Indel	PV	10	c.1961delA	p.Lys654fs	-	4	4
	Chr17:41256250	rs80357604	7	Indel	PV	5	c.329delA	p.Lys110fs	-	5	2
	Chr17:41243479	rs80357508	1	Indel	PV	10	c.4065_4068delTCAA	p.Asn1355fs	-	-	1
*VHL*	Chr3:10188296	rs869025653	12	Indel	PV	2	c.444delT	p.Phe148fs	2	8	2
*MSH6*	Chr2:48030691	rs267608092	6	Indel	PV	5	c.3312delT	p.Phe1104fs	1	4	1
	Chr2:48025764	rs1800937	2	SNP	PV	3	c.642C > A	p.Tyr214Ter	1	1	-
*APC*	Chr5:112175100	rs587783033	6	Indel	PV	15	c.3814delT	p.Ser1272fs	1	3	2
	Chr5:112173393	rs587783030	1	Indel	PV	15	c.2107delG	p.Ala703fs	-	1	-
*CHEK2*	Chr22:29099524	rs772683219	7	Indel	PV	9	c.1005delT	p.Phe335fs	1	4	2
*MSH2*	Chr2:47709924	rs63750084	6	Indel	PV	16	c.2647delA	p.Ile883fs	-	5	1
*MEN1*	Chr11:64572092	rs794728642	1	Indel	PV	10	c.1561delC	p.Arg521fs	-	-	1
*PALB2*	Chr16:23646857	rs45494092	1	SNP	PV	4	c.1010T > A	p.Leu337Ter	-	-	1
*MUTYH*	Chr1:45797228	rs36053993	1	SNP	LPV	13	c.1187G > A	p.Gly396Asp	-	1	-

HGVS.c: Human Genome Variation Society, Coding DNA sequence; HGVS.p: Human Genome Variation Society, protein sequence; Chr.: chromosome. PV: pathogenic variants; LPV: likely pathogenic variants; VUS: variants of uncertain significance; *: indicate a translation termination (stop) codon.

**Table 3:** Prevalence of germline mutations in DNA repair genes and among different tumor grades.

DNA Repair Genes			Mutation Cases (Out of 101)	Prevalence (%)	Grade I (%)	Grade II (%)	Grade III (%)	*p*-Value
**Double strand repair**	**HRR**	*BRCA1*	20	19.8		14 (70)	6 (30)	0.42
		*BRCA2*	31	30.7	2 (6.5)	21 (67.7)	8 (25.8)	
		*PALB2*	1	0.99			1 (100)	
Single strand repair	MMR	*MSH2*	6	5.6		5 (83.3)	1 (16.7)	0.69
		*MSH6*	8	7.9	2 (25)	5 (62.5)	1 (12.5)	
	BER	*MUTYH*	1	0.99		1 (100)		
Checkpoint		*ATM*	31	30.7	1 (3.22)	17 (54.84)	13 (41.94)	0.45
		*CHEK2*	7	6.9	1 (14.29)	4 (57.14)	2 (28.57)	0.42

BER: base excision repair; HR: homologous recombination repair; MMR: mismatch repair; BC: breast cancer.

**Table 4:** List of patient numbers that carried pathogenic germline mutations in single and multiple genes.

**Gene**	**Affected Transcript**	**Patient No.** **(Alteration in 1 Gene)**	**Patient No.** **(Alteration in 2 Genes)**	**Patient No. ** **(Alteration in 3 Genes)**	**Patient No.** **(Alteration in 4 Genes)**	**Patient No.** **(Alteration in 5 Genes)**	**Patient No.** **(Alteration in 6 Genes)**
***BRCA1***	c.1961delA		Sample 6 *	Sample 1 * Sample 22 Sample 36	Sample 86 *	Sample 70 *	Sample 87 *
			Sample 34				
	c.329delA		Sample 30 *	Sample 82	Sample 86 *	Sample 70 *	Sample 87 * Sample 90
			Sample 15				
	c.4065_4068delTCAA		Sample 30 *				
	c.3214delC	Sample 16 Sample 17 Sample 7 Sample 51	Sample 6 * Sample 21 Sample 25 Sample 28	Sample 1 * Sample 32			
***BRCA 2***	c.7543delA	Sample 72 Sample 97	Sample 6 * Sample 15 Sample 78 Sample 98	Sample 82 Sample 71	Sample 86 Sample 79 Sample 84	Sample 70 * Sample 55 Sample 89	Sample 87 * Sample 90 * Sample 66
	c.3860delA	Sample 20 * Sample 42		Sample 1 * Sample 32 Sample 4 Sample 36		Sample 70 *	Sample 90 * Sample 87 *
	c.9097delA	Sample 20 * Sample 41 Sample 39	Sample 6 * Sample 34 Sample 29 Sample 43 Sample 50				
	c.5073delA					Sample 70 *	
	c.4808delA					Sample 70 *	Sample 87 *
	c.5291C > G	Sample 33					
	c.5763delT			Sample 1 *		Sample 70 *	
	c.774_775delAA	Sample 8					
***ATM***	c.8431_8432delAA	Sample 19 Sample 53 Sample 35 Sample 96 * Sample 104 *	Sample 21 * Sample 30 Sample 25 Sample 28 Sample 29 Sample 43 Sample 94	Sample 1 Sample 32 * Sample 99 * Sample 22 Sample 71 Sample 4 Sample 36	Sample 79 Sample 84	Sample 55 Sample 89	Sample 87
	c.2754delT	Sample 56	Sample 21 * Sample 60 *	Sample 32 * Sample 99 *			Sample 66
		Sample 64					
		Sample 77 Sample 96 *					
		Sample 101 Sample 104 *					
	c.478_482delTCTCA		Sample 21 *				
	c.3526delC		Sample 50				
	c.5932G > T		Sample 60 *				
***VHL***	c.444delT		Sample 83 Sample 98	Sample 82 Sample 71	Sample 86 Sample 79 Sample 84	Sample 55 Sample 89	Sample 87 Sample 90 Sample 66
***MSH6***	c.642C > A	Sample 38		Sample 4			
	c.3312delT		Sample 60	Sample 99	Sample 86	Sample 55 Sample 89	Sample 66
***APC***	c.3814delT	Sample 58 Sample 65	Sample 83			Sample 70	Sample 90 Sample 66
	c.2107delG	Sample 48					
***CHEK2***	c.1005delT		Sample 78 Sample 94		Sample 84	Sample 70 Sample 55 Sample 89	Sample 90
***MSH2***	c.2647delA			Sample 99	Sample 79	Sample 70	Sample 87 Sample 90 Sample 66
***MEN1***	c.1561delC						Sample 87
***PALB2***	c.1010T > A			Sample 22			
***MUTYH***	c.1187G > A	Sample 102					

* Patients with more than one mutation (multi-hit) within the same gene.

**Table 5:** List of variants of uncertain significance and variants of conflicting interpretation of pathogenicity identified with multi-gene panel sequencing.

dbSNP	Gene Name	Clinical Significance	dbSNP	Gene Name	Clinical Significance
rs146297864	NEK2	VUS	rs730881396	AXIN2	VUS
rs1799939	RET	Conflicting interpretations of pathogenicity	rs28997569	BRIP1	Conflicting interpretations of pathogenicity
rs607969	MEN1	Conflicting interpretations of pathogenicity	rs367696886	XRCC2	VUS
rs2229022	ATM	Conflicting interpretations of pathogenicity	rs372305287	APC	Conflicting interpretations of pathogenicity
rs144636562	ATM	VUS	rs371264852	STK11	Conflicting interpretations of pathogenicity
rs770406711	APC	VUS	rs373226409	MSH2	Conflicting interpretations of pathogenicity
rs138743097	BRIP1	VUS	rs4988345	BRIP1	Conflicting interpretations of pathogenicity
rs138749920	RAD50	VUS	rs587782683	MUTYH	VUS
rs138933660	APC	Conflicting interpretations of pathogenicity	rs62625284	PALB2	Conflicting interpretations of pathogenicity
rs777004819	MER11A	VUS	rs786203658	NF1	VUS
rs141142822	ERBB2	VUS	rs751431238	MSH2	VUS
rs145415033	MER11A	VUS	rs759105985	PALID	VUS
rs149342980	MUTYH	VUS	rs761673463	BARD1	VUS
rs149815077	GEN1	VUS	rs180727534	SYNE1	VUS
rs189059377	BMPR1A	VUS	rs201707558	PALLD	VUS
rs273901741	BRCA1	Conflicting interpretations of pathogenicity			

VUS: variants of uncertain significance.

## Data Availability

The data generated during this study are included in this article.

## Supplementary Materials

The following supporting information can be downloaded at: https://www.mdpi.com/article/10.3390/genes14010106/s1, Supplementary File S1: The whole set of the identified variants in this study.
